# Innovation diffusion process of higher education informatization based on Lotka-Volterra model

**DOI:** 10.1371/journal.pone.0325687

**Published:** 2025-07-28

**Authors:** Junjuan Liu, Peiling Yan

**Affiliations:** School of Information Technology, Henan University of Chinese Medicine, Zhengzhou, China; Federal University of Technology - Parana, BRAZIL

## Abstract

In order to quantify the diffusion speed, influence scope and change trend of educational informatization innovation in colleges and universities, this paper studies the diffusion process of educational informatization innovation in colleges and universities based on Lotka-Volterra model. A research has constructed an framework that includes system input, processing, upgrading, and output: in the system input stage, discourse analysis methods are used to reveal the antecedents of educational informatization innovation; During the system processing phase, analyze the interaction between these antecedents to clarify the mechanism of innovation implementation; During the system upgrade phase, the Lotka Volterra model is integrated to quantitatively analyze the dynamic characteristics of innovation diffusion; In the system output stage, evaluate the specific impact of innovation diffusion on teaching content, teaching forms, and other aspects based on the analysis results. The empirical results show that there is a positive correlation between the competition coefficient and the innovation diffusion effect of university education informatization; Narrowing the gap in the diffusion speed of educational informationization innovation among colleges and universities can promote the balanced development of educational informationization and improve the effect of educational informationization innovation diffusion in colleges and universities; The Cronbach’s α values of teaching content, teaching form and other impact type variables are higher than 0.7, indicating that the impact of the diffusion of information technology in higher education on these aspects is stable and reliable.

## 1 Introduction

As the cradle of talent cultivation and the forefront of scientific research and innovation [1], the process of informatization in education not only concerns the improvement of education quality, but also directly affects the country’s innovation ability and international competitiveness. The rapid development of information technology, especially the Internet, big data, artificial intelligence, the Internet of Things and other technologies are widely used, for the field of education has brought unprecedented opportunities and challenges [2]. Education informatization is no longer just a technological innovation, but a profound change in the concept of education, teaching mode, learning mode and even education ecology [3]. It allows educational resources to cross the time and space limitations, to achieve optimal allocation and sharing, and to promote the double enhancement of educational equity and quality [4]. In the wave of education informatization, colleges and universities, as the core component of the education system, bear the multiple missions of cultivating high-level talents, promoting scientific and technological innovation, and serving economic and social development. Therefore, university education informatization is not only an inevitable requirement of education modernization, but also a key way to enhance the comprehensive competitiveness of universities. The diffusion process of education informatization in colleges and universities refers to the introduction of new education information technology, concepts and methods in colleges and universities from the introduction to the process of wide application. This process not only involves the dissemination and adoption of technology, but also involves changes in educational concepts, teaching models, management styles and other aspects [5]. Therefore, under the background of information technology, the traditional teaching mode is teacher-centered and classroom lecturing-based, which is difficult to meet the individualized and diversified learning needs of students. Through in-depth study of the communication process of educational information technology innovation, college educators can be guided to establish student-centered, capacity-cultivating and practice-innovation-centered educational concepts, thus promoting the modernization of educational concepts.

By analyzing the process and law of innovation diffusion of educational informatization in colleges and universities, this paper reveals its internal mechanism, provides theoretical basis for the formulation and implementation of educational informatization policies, and improves the quality of education. For example, Hooda and others studied how to use facts to obtain more academic learning and improve students’ overall performance. Through technology authorization, teachers can provide timely and effective feedback to achieve better learning. Through these studies, it is noted that negative feedback will hinder learners’ efforts and achievements, so it should be carefully designed and delivered. In this work, a new method based on improved FCN (full connection network) is designed. The key of this method is to standardize the quality assessment of higher education students. The proposed method consists of different stages: the first stage is data acquisition, in which data are collected from various sources for training and testing the proposed method. The second stage is data oriented, where information is oriented in a specific file format. Then, the data is cleaned and preprocessing methods are applied. In the fourth stage, develop a machine learning based model to predict students’ learning achievements and evaluate the quality of students after the innovation of higher education [6]. The performance of FCN is highly dependent on the quality and quantity of input data. In the evaluation of students’ quality in higher education, if the data is missing, noisy or inconsistent, it will directly affect the accuracy and stability of the prediction model. Sitar-Taut, D. A and others established a new comprehensive model, SD-UTAUT (social distance – utaut), on the basis of the extended unified theory of technology acceptance and use, to better understand the relationship between the original structure and university innovation ability (PI) and information quality (IQ). The key factors influencing behavioral intention (BI) and use were identified by examining the mediating effects of revaluation of hedonic motivation (HM) and learning value (LV). The results show that IQ has the greatest impact on LV, HM has greater impact on use behavior (UB) than LV, but has the greatest impact on habit (HT). The fun and pleasure of using mobile learning will stimulate LV through interesting and interactive content [7]. Although SD-UTAUT model can better describe various factors and their relationships in the process of technology acceptance and use, it has certain limitations in dynamic simulation and long-term prediction. The prediction ability of the model is affected by factors such as data quality, model assumptions and changes in the external environment. Comsa and others proposed a resource allocation solution based on machine learning to realize the innovation of college education, which can improve the quality of distance education to meet the needs of more and more scholars. This solution is deployed and tested in the university education environment. Compared with the existing technology, it proves the advantages in the main service quality parameters of various distance education contents, and provides reference for the innovation and expansion of university education [8]. The performance of machine learning model is highly dependent on the quality and integrity of data. If the data is biased or missing, it will affect the accuracy of model prediction and the rationality of resource allocation. Mohaisen and others improved the connectivity of the education network, promoted the diffusion of innovative knowledge, and objectively evaluated the educational achievements by strengthening the relationship between students and teachers and students. First, students are divided into guidance groups, each group has a homework. Then, the task is solved for the tutor student, so the non-tutor (tutor) student should discuss with at least one tutor student. Finally, the students reported on their friendship with each other when they submitted their homework. The scores ranged from 0 to 4. In addition, tutor students report the number of interactions with non tutor students and the duration of each interaction. The results show that the collected data points are analyzed using social network analysis tools, and impressive improvements have been made in the quantity and quality of relationships. Compared with that at the beginning of the semester, the local characteristics based classification and mixing are dominant, and the number of friendships established between students through network connections has increased by 20 times. The participation of students in the social learning environment can improve the quantity and quality of the relationship between students and teachers, and is conducive to the diffusion of innovative knowledge [9]. Although the method of strengthening the relationship between students can improve the connectivity of the education network, its ability to predict future student behavior, relationship development and knowledge diffusion is relatively weak. It depends more on the current practice effect and experience summary than strict mathematical model prediction. Jiang L et al. conducted semi-structured interviews with 18 EFL students and 4 teachers, qualitatively analyzed the data using NVivo 11 software, and constructed an “Environment Human Mediated Behavior” (EPMB) model through three-layer coding analysis to elucidate the mechanisms influencing deep learning among EFL students in HVCs. The research results indicate that intrinsic motivation and cognitive ability are crucial for EFL students’ deep learning in HVCs; The blended learning environment, English course satisfaction, and English teacher teaching methods are situational influencing factors that are interrelated and have positive or negative effects on deep learning through various mediators [10]. However, this method lacks systematicity and coherence in the research of each stage, which is not conducive to a comprehensive and in-depth analysis of the diffusion process of educational informatization innovation. Huang et al. proposed a robust natural language processing model based on the BERT Bi LSTM framework, trained on 11226 manually labeled posts, for classifying educational technology course forum posts. Multiple regression analysis shows that information support is negatively correlated with emotional exhaustion and inappropriate behavior, while emotional support is negatively correlated with emotional exhaustion and low sense of achievement. The moderation effect analysis shows that self-regulated learning moderates the negative correlation between information support and inappropriate behavior, emotional support and emotional exhaustion, and has a stronger impact on learners with low self-regulated learning levels [11]. But this method mainly reveals the correlation between variables and does not simulate and predict the dynamic changes of the research object.

In summary, various models have been proposed to study the diffusion process of innovation in the field of education, but these models have limitations in dynamic simulation and long-term prediction, and their predictive ability is affected by multiple factors. And this study chose the Lotka Volterra model because it is an important model in biological mathematics [12], which can effectively characterize the evolution and development of populations (similar to the diffusion of educational information technology in universities) in specific environments [13]. Through the mathematical model, we can quantify the diffusion speed, influence range and change trend of educational informatization innovation in colleges and universities, and provide accurate data support for decision makers. At the same time, this model can simulate the diffusion process of educational informatization innovation among different colleges and universities, and consider the impact of various internal and external factors (such as policy support, capital investment, technology maturity, teacher acceptance, etc.) on the diffusion process. The dynamic simulation helps to predict the future development trend and provides a scientific basis for strategic planning. Therefore, this paper studies the innovation diffusion process of university education informatization based on Lotka-Volterra model to better understand the nature and law of education informatization and provide strong support for the in-depth development of university education informatization. The main contributions of this study are as follows:

(1) Building a new analytical framework: constructing an analytical framework that includes system input, processing, upgrading, and output, comprehensively analyzing the process of innovation and diffusion of information technology in higher education.
(2) Quantify key relationships: Use the Lotka Volterra model to quantitatively analyze the dynamic characteristics of innovation diffusion, clarify the diffusion speed, impact range, and change trend.
(3) Revealing internal mechanisms: Through methods such as discourse analysis and interaction analysis, reveal the antecedents and implementation mechanisms of educational informatization innovation.
(4) Assessing specific impacts: Based on the analysis results, evaluate the specific impacts of innovation diffusion on teaching content, teaching forms, and other aspects, and provide targeted suggestions for the development of information technology in higher education.

## 2 Analysis of the diffusion process of innovations in higher education informatization

### 2.1 Framework for analyzing the diffusion process of innovation in higher education informatization

**Fig 1:**
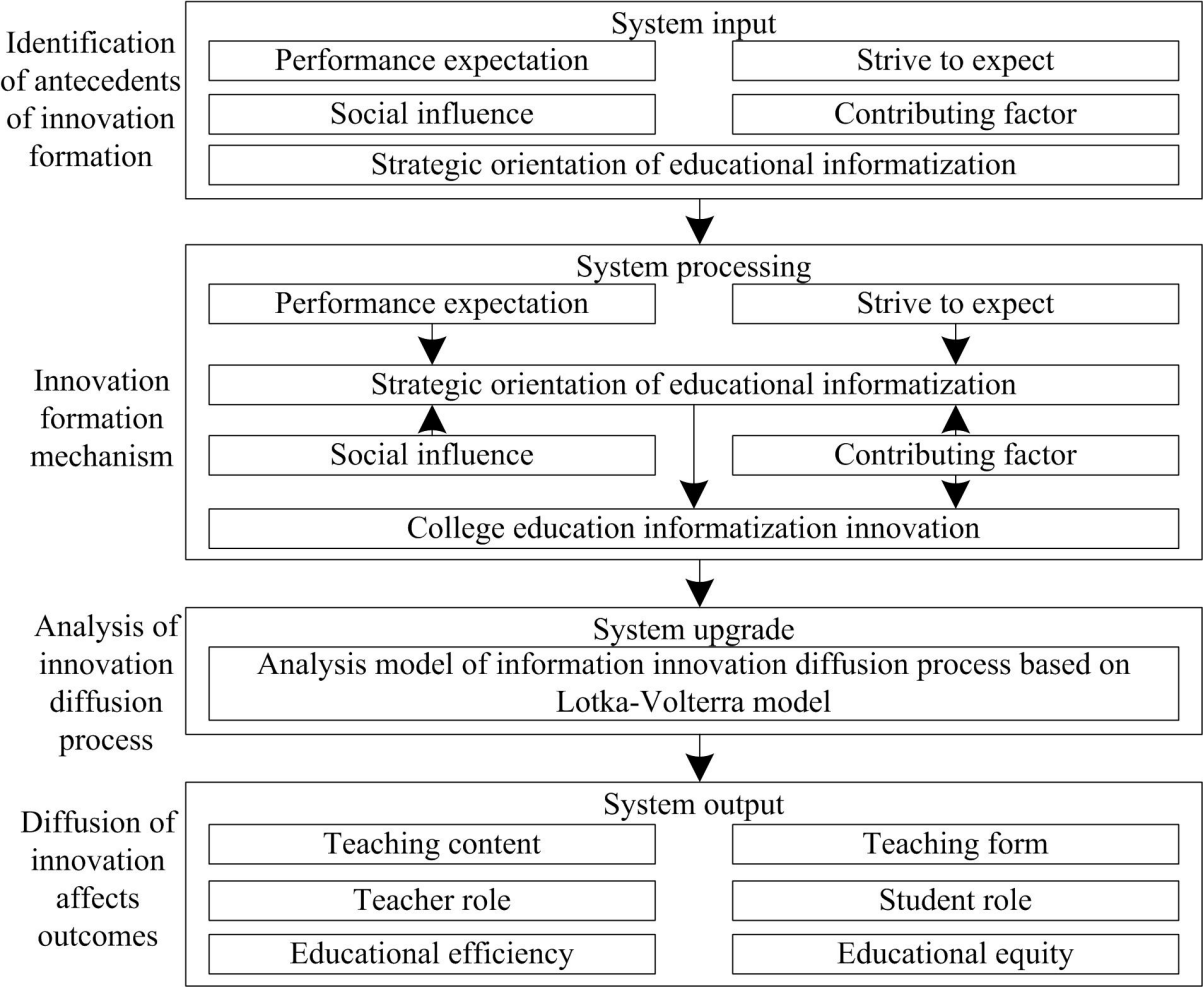
Analysis framework of the diffusion process of college education informatization innovation.

The analysis of the diffusion process of education informatization innovation in colleges and universities can draw on the system logic, which can be regarded as a dynamic system consisting of four parts: input, processing, upgrading and output. This process not only promotes the internal teaching and management efficiency of colleges and universities [14], but also outputs the sustainability effects of education quality improvement, social value enhancement and optimal allocation of educational resources. The analytical framework of the diffusion process of educational informatization innovation in colleges and universities is shown in Fig 1.

(1) System input: the sowing of innovation “seeds”. In the early stage of college education informatization innovation, it is necessary to identify and sow the “seeds” of innovation, that is, the antecedents of innovation formation. These antecedents include.
  a. Performance expectations: including the expected improvement of teaching quality [15], improvement of students’ learning effect, improvement of management efficiency and other dimensions. These expectations drive colleges and universities to seek solutions for education informatization.
  b. Effort expectation: it involves teachers’ acceptance of new technologies, the effectiveness of technical training, the adequacy of technical support, etc., which affects the difficulty of implementing educational informatization in colleges and universities.
  c. Social impact: it includes policy promotion, peer demonstration, suggestions from education experts, etc. These external factors promote universities to adopt educational informatization innovation.
  d. Contributing factors: contributing factors include financial support, technical infrastructure, leadership support, etc. These resource conditions provide the basis for educational informatization innovation.
  e. Strategic orientation of education informatization: the planning and vision of universities for the long-term development of education informatization directly affect the direction and depth of innovation.
(2) System processing: the convergence of innovation “streams”. After the innovation “seed” is sown, different antecedents interact with each other to form specific mechanisms and paths (i.e., “streams”) to promote the implementation of education informatization innovation. The interaction between these antecedents does not exist in isolation, but is interrelated and mutually influential, jointly promoting the advancement of educational informatization innovation. The specific mechanisms include:
  a. The strategic orientation of educational informatization directly drives the initiation and implementation of innovative projects. At the same time, it provides direction and framework for other antecedents to play a role. For example, performance expectations are set based on the strategic orientation of educational informatization, which clarifies the long-term development plan and vision of higher education informatization, providing specific directions for performance expectations, including the expected improvement of teaching quality and management efficiency under specific strategic directions.
  b. Performance expectations indirectly affect the selection and promotion of innovative projects through the strategic orientation of education informatization. On the one hand, performance expectations prompt universities to seek educational informatization solutions to meet these expectations, while the strategic orientation of educational informatization guides universities to choose projects that align with the strategic direction among numerous solutions. On the other hand, when performance expectations align with strategic orientation, it strengthens the driving effect of strategic orientation on innovative projects and promotes smoother project progress. If the strategic orientation emphasizes the use of information technology to improve teaching quality, and performance expectations also focus on improving teaching quality, then when choosing innovative projects, it will be more inclined to those that can directly promote the improvement of teaching quality.
  c. Efforts are expected to indirectly promote the deepening of educational informatization by improving teachers’ ability to master and apply technology. Factors such as the strategic orientation of educational informatization and social influence will have an impact on the expected effort. If the strategic orientation of educational informatization emphasizes teacher training and technical support, it will enhance teachers’ acceptance of new technologies and increase their expectations for effort. Peer demonstrations and expert advice in social impact can also enable teachers to see the positive effects brought by new technologies, thereby increasing their expectations for effort. When universities see other peers improving teaching effectiveness through the adoption of new technologies, teachers will be more willing to learn and apply new technologies, thereby increasing their expectations and promoting the deepening of educational informatization.
  d. Social influence stimulates the internal innovation power of colleges and universities through the display and sharing of external successful cases [16]. Meanwhile, social influence will also interact with the strategic orientation of educational informatization. As a part of social influence, policy promotion will guide universities to adjust their strategic orientation, making it more in line with policy requirements and development trends. The clear strategic orientation will also enable universities to pay more targeted attention to relevant information in society and better utilize external successful cases to stimulate internal innovation momentum. When policies encourage universities to innovate in online teaching, they will adjust their strategic orientation, pay more attention to successful cases of online teaching in other universities, draw experience from them, and stimulate their own innovation motivation.
  e. Contributing factors not only provide basic support for educational informatization innovation, but also may directly trigger the development of innovation activities. Financial support, technological infrastructure, and other contributing factors can affect the formulation and implementation of educational informatization strategies. Adequate funding and well-developed infrastructure can ensure the smooth implementation of strategic orientation, and strategic orientation will also guide the rational allocation of contributing factors. If the strategic orientation emphasizes the development of virtual reality teaching, then universities will tilt towards this field in terms of funding allocation and infrastructure construction to promote the development of related innovative activities.

By analyzing the convergence process of these “streams”, we can clarify the implementation mechanism of educational informatization innovation in universities, understand how different antecedents interact and promote each other, and jointly promote the implementation and development of educational informatization innovation in universities.

(3) System upgrading: diffusion of innovation “wave”. The upgrading of educational informatization innovation is realized through its diffusion among universities. Based on the implementation mechanism of university education informatization innovation, the Lotka Volterra model is used to analyze the diffusion process of university education informatization innovation, forming a “wave” of innovation.
  a. Growth of diffusers: the diffusion speed of educational informatization innovation is jointly affected by the enhancement of its own influence and the acceptance of colleges and universities. When colleges and universities actively adopt and promote educational informatization innovation, the impact of innovation (i.e., the number of “predators”) will increase. This process can be expressed as the growth function of the number of diffusers, part of which comes from the positive feedback brought by universities’ acceptance and successful application of innovation [17].
  b. Change in college acceptance: college acceptance of educational informatization innovation (i.e., the number or status of “predators”) is affected by the speed of innovation diffusion, policy guidance, resource support, and exchanges and cooperation between colleges and universities [18]. When innovation achievements are significant, policies are favorable, resources are sufficient and communication is frequent, universities are more inclined to accept and adopt educational informatization innovation.
  c. Interaction: The interaction between educational informatization innovation and universities is similar to the dynamic balance between predators and prey. On the one hand, the diffusion of innovation promotes the acceptance and application of universities; On the other hand, the positive feedback of colleges and universities accelerates the further diffusion and upgrading of innovation.
(4) System output: the harvest of the “fruit”. The ultimate goal of college education informatization innovation is to improve the quality of teaching, promote student development, optimize management efficiency, etc. [19]. Through the analysis of the innovation diffusion process of educational informationization in colleges and universities, the influence of the innovation diffusion of educational informationization in colleges and universities on teaching content, teaching form, teachers’ role, students’ role, educational efficiency and educational equity can be evaluated, so as to provide suggestions for further optimizing the innovation of educational informationization and promote the development of the system to a more positive cycle [20].

### 2.2 Identification of antecedents for the formation of innovation in higher education informatization

The input part of the system uses discourse analysis method to identify the antecedents of the formation of university education informatization innovation, and among 1384 historical discourses related to university education informatization innovation, discourse refinement is carried out to sort out the discourse evidences reflecting the relationship between the main categories, and the story line between the main categories is extracted, as shown in Table 1.

**Table 1:** Reflects the original discourse of the story line.

Story line	Discourse evidence
Strategic orientation of education informatization → Willingness to adopt innovation	The recognition of the importance of educational informatization by university management is the primary driving force to promote its adoption and innovative application. The recognition of the potential of technology promotes the exploration and attempt in the field of education informatization.
Performance expectation → educational informatization innovation adoption intention → innovation implementation	The expected tangible and intangible benefits such as the improvement of education quality and the increase of teaching efficiency are the key factors for universities to consider the adoption of education informatization innovation. These expected benefits strengthen the determination of universities to promote the implementation of innovation.
Effort expectation → educational informatization innovation adoption intention → innovation implementation	The confidence of teachers and students in mastering technology and the perfection of school technology infrastructure are important factors to determine whether the innovation of education informatization can be effectively adopted and implemented.
Social impact → Willingness to adopt educational informatization innovation → Innovation implementation	Successful cases inside and outside the industry, policy orientation and the influence of society’s positive evaluation of education informatization have prompted colleges and universities to adopt education informatization innovation to keep up with the pace of the times and enhance competitiveness.
Contributing factors → Willingness to adopt educational informatization innovation → Innovation implementation	Existing experience with education informatization projects, partnership opportunities with industry leaders, and continued funding provide universities with the necessary support and resources to help them determine the direction of innovation and develop feasible implementation plans.

In Table 1, within the 1384 original discourse statements, there are 273 statements reflecting “strategic orientation of education informatization→willingness to adopt innovations”; 307 statements reflecting “performance expectations→willingness to adopt innovations in education informatization→innovation implementation”; 283 statements reflecting “strive expectation→willingness to adopt innovations in education informatization→innovation implementation”; and 274 statements reflecting “social influence→willingness to adopt innovations in education informatization→innovation implementation”; and 247 statements reflecting the “contributing factor→willingness to adopt innovations in education informatization→innovation implementation”. For this reason, strategic orientation of education informatization, performance expectation, effort expectation, social influence, and contributing factors are identified as the antecedents of the formation of education informatization innovation in universities.

### 2.3 Diffusion process analysis hypothesis of innovation diffusion in higher education informatization

Assuming that the whole process of diffusion of education informatization innovations in colleges and universities is carried out in a stage-by-stage manner, the result of innovation adoption depends on the acceptance and implementation ability of each college and university [21]. Colleges and universities that fail to adopt the innovation will not be able to continue to participate in the innovation activities in the next stage, while successful adoption of the innovation of colleges and universities will enter a deeper level of application and optimization stage. Because there are many uncontrollable human and environmental factors in the process of university education informatization, and these factors are often difficult to quantify, therefore, in constructing the interaction model of diffusion of informatization innovations, the following hypotheses are proposed.

(1) Stability assumption of informatization resources: Before and after the diffusion of educational informatization innovations, the total amount of informatization resources in colleges and universities remains unchanged, which ensures the comparability of the analysis of the diffusion process.
(2) Diffusion process independence hypothesis: the diffusion results of educational informatization innovations are independent of other non-normal factors, and are mainly influenced by internal and external normal factors of universities.
(3) Competition and cooperation impact hypothesis: in the process of diffusion of education information technology innovations, both competition and cooperation between universities will affect the diffusion rate of innovations, competition may slow down the process of each other’s innovations, while cooperation may accelerate the diffusion of innovations in both universities.
(4) Group interaction hypothesis: the diffusion of informatization innovation is not only reflected in the level of a single university, but also in the interaction between university groups. Competition and cooperation between groups have a significant impact on innovation diffusion.

### 2.4 Construction of analytical model for innovation diffusion process of university education informatization based on Lotka Volterra model

Lotka-Volterra model can quantify the diffusion speed, influence scope and change trend of educational informatization innovation in colleges and universities, and simulate the diffusion process of educational informatization innovation among different colleges and universities [22], considering the impact of various internal and external factors (such as policy support, capital investment, technology maturity, teacher acceptance, etc.) on the diffusion process. Therefore, based on the clear implementation mechanism of university education informatization innovation in the system processing part, the Lotka-Volterra model is used to analyze the diffusion process of university education informatization innovation. In the analysis process, the university groups that have achieved informatization innovation (can be regarded as “diffusers”) and the university groups that are adopting or are about to adopt informatization innovation (can be regarded as “adopters”) are usually analyzed. Under the competition relationship, the interaction coefficient in Lotka-Volterra model represent the competition intensity between the two groups. If there is a cooperative relationship between implementers and adopters, the interaction coefficient will indicate the cooperation intensity.

The classic Malthus model is formula (1):


$$\left\{ \;\;\;\begin{array}{c} \frac{dM\left(t\right)}{dt}=rM\left(t\right)\\ M\left(t_{0}\right)=M_{0} \end{array}\right.$$ (1)

In formula (1), M(t) is the number of college groups at time t, which is used to describe how the number of college groups has evolved over time during the diffusion of educational informatization. r is the intrinsic diffusion velocity; $t_{0}$ is the initial moment; $M_{0}$ is the number of initial college groups.

The logistic growth model shown in formula (2) can be obtained from formula (1):


$$\left\{ \;\;\;\;\begin{array}{c} \frac{dM\left(t\right)}{dt}=rM\left(t\right)\left(1-\frac{M\left(t\right)}{K}\right)\\ M\left(t_{0}\right)=M_{0} \end{array}\right.$$ (2)

In formula (2), K represents the maximum environmental capacity. The largest scale or level of development that can be supported by the university education informatization system or practice under the constraints of existing resources and environmental conditions. It is precisely because of the introduction of this new term, which promotes the progress of human research. As the research progresses, the survival problem of multiple groups in the same range is studied, and the existence of one group is found to inhibit the development of other groups. At the same time, there is resource competition among multiple groups, so the classic Lotka-Volterra competition model emerges, as shown in formula (3):


$$\left\{ \;\;\;\;\begin{array}{c} \frac{d\rho_{1}}{dt}=r_{1}\rho_{1}\left(1-\frac{\rho_{1}+c_{1}\rho_{2}}{{\rho_{1,}}_{\max}}\right)\\ \frac{d\rho_{2}}{dt}=r_{2}\rho_{2}\left(1-\frac{\rho_{2}+c_{2}\rho_{1}}{{\rho_{2,}}_{\max}}\right) \end{array}\right.$$ (3)

In formula (3), $\rho_{1}$ and $\rho_{2}$ represent the density of informational education knowledge in the two college populations, respectively; ${\rho_{1,}}_{\max}$ and ${\rho_{2,}}_{\max}$ represent the maximum density of informational education knowledge for the two college groups, respectively; $r_{1}$ and $r_{2}$ represent the intrinsic diffusion rates of the two college populations, respectively. $c_{1}$ and $c_{2}$ represent the interaction coefficients of the two college groups, respectively.

The Lotka-Volterra competition model of formula (3) is deduced and defined $\frac{M\left(t\right)}{K}$ as the knowledge density ρ of the university group at time t. It is easy to know that as the density increases, the growth rate of the university community will gradually slow down under the existing resources and environmental conditions. In the process of diffusion of educational informatization. $\frac{m\left(t\right)}{M\left(t\right)}$ indicates the instantaneous growth rate of the number of college groups in time t, which is used to measure the speed of diffusion of education informatization in colleges and universities, reflecting the spreading efficiency and influence of education informatization in the college and university community. m(t) is the derivative of M(t) about time t. r is the constant rate of diffusion of the college population under the constraints of existing resources and environmental conditions; If K tends to infinity, then the number of college groups at equilibrium will grow exponentially with a constant instantaneous growth rate, i.e., formula (4).


$$\frac{m\left(t\right)}{M\left(t\right)}=r\left(1-\frac{M\left(t\right)}{K}\right)$$ (4)

In fact, K is always finite. If the instantaneous rate of growth of the college population is assumed to be a linear function of the density of the college population, combined with the constraints on the size of the college population, it is assumed to be formula (5):


$$\frac{m\left(t\right)}{M\left(t\right)}=r\left(1-\frac{M\left(0\right)\exp\left(rt\right)}{K}\right)$$ (5)

Solve formula (6):


$$M\left(t\right)=\frac{KM\left(0\right)\exp\left(rt\right)}{K-M\left(0\right)+M\left(0\right)\exp\left(rt\right)}$$ (6)

The equilibrium solution under the first order condition is formula (7):


M(t)=K (7)

In the process of diffusion of innovations in educational informatization in the above mentioned universities another group of universities is introduced, forming a situation where two groups of universities exist in the same system of educational informatization in the same university. In the text, $M_{1}\left(t\right)$ and $M_{2}\left(t\right)$ denotes two university education informatization innovation groups in the university education informatization system. Obviously, if there is no competition, the growth process of the two university education informatization innovation groups $M_{1}$ and $M_{2}$ is consistent with the above process, and will reach their respective maximum capacities $K_{1}$ and $K_{2}$, i.e., the number of each of the two university education informatization innovation groups remains stable at that equilibrium point.

Considering the influence of the competitive exclusion effect produced by the existence of college education informatization innovation group $M_{2}$ on $M_{1}$, according to the previous linear assumption, the instantaneous growth rate of the combined formula (3) $M_{1}$ can be expressed as formula (8):


$$\frac{m_{1}\left(t\right)}{M_{1}\left(t\right)}=r_{1}\left(1-\frac{M_{1}\left(t\right)+c_{2}M_{2}\left(t\right)}{K_{1}}\right)$$ (8)

In formula (8), $-\frac{c_{2}M_{2}\left(t\right)}{K_{1}}$ denotes the inhibitory effect of the college education informatics innovation community $M_{2}$ diffusion of innovation on $M_{1}$ (c denotes the coefficient of competition), where there is $0<c_{2}<1$, or $c_{2}>1$, respectively, indicate that in the process of diffusion of innovations in educational informatization in higher education. The diffusion effect of $M_{2}$ education informatization innovation is weaker than or stronger than the diffusion effect of $M_{1}$, (the higher the value of the competition coefficient, the greater the deterrent effect on competitors). The meanings expressed for $c_{1}$ is similar.

Similarly, in combination with formula (3), the instantaneous growth rate of $N_{2}$ can be expressed as formula (9):


$$\frac{m_{2}\;\left(t\right)}{M_{2}\;\left(t\right)}=r_{2}\left(1-\frac{M_{2}\;\left(t\right)+c_{1}M_{1}\;\left(t\right)}{K_{2}}\right)$$ (9)

When the education informatization innovation diffusion speed of the two university groups is 0, it indicates that the education informatization innovation diffusion reaches equilibrium, and the education informatization innovation field tends to be saturated with the acceptance of education informatization innovations, and the formula (10) is established:


$$\left\{ \;\;\;\begin{array}{c} 1-\frac{M_{1}\left(t\right)}{K_{1}}-\frac{c_{2}M_{2}\left(t\right)}{K_{1}}=0\mathrm{\backslash vspace}1mm\\ 1-\frac{M_{2}\left(t\right)}{K_{2}}-\frac{c_{1}M_{1}\left(t\right)}{K_{2}}=0 \end{array}\right.$$ (10)

The equilibrium conditions of the diffusion and evolution of educational informatization innovation are expressed as formula (11) composed of $m_{1}\left(t\right)$ and $m_{2}\left(t\right)$:


$$\left\{ \;\;\;\begin{array}{c} f_{1}\left(m_{1}\left(t\right),m_{2}\left(t\right)\right)=1-\frac{M_{1}\left(t\right)}{K_{1}}-\frac{c_{2}M_{2}\left(t\right)}{K_{1}}\mathrm{\backslash vspace}1.5mm\\ f_{2}\left(m_{1}\left(t\right),m_{2}\left(t\right)\right)=1-\frac{M_{2}\left(t\right)}{K_{2}}-\frac{c_{1}M_{1}\left(t\right)}{K_{2}} \end{array}\right.$$ (11)

Diffusion evolution trajectories on both sides using straight lines $f_{1}\left(m_{1}\left(t\right),m_{2}\left(t\right)\right)=0$ and $f_{2}\left(m_{1}\left(t\right),m_{2}\left(t\right)\right)=0$ to express, taking into account the actual situation of the proliferation of innovations in the informatization of education in higher education, only $m_{1}\left(t\right)$ and $m_{2}\left(t\right)$ are all greater than 0, there has practical significance, i.e., the straight line has practical value only in the first quadrant. When $f_{1}\left(m_{1}\left(t\right),m_{2}\left(t\right)\right)=0$ and $f_{2}\left(m_{1}\left(t\right),m_{2}\left(t\right)\right)=0$, solving the system of formulas yields three equilibrium points, $A_{1}\left(M_{1}\left(t\right),0\right)$, $A_{2}\left(0,M_{2}\left(t\right)\right)$, $A_{3}\left(\frac{\left(1-c_{2}\right)M_{1}\left(t\right)}{1-c_{1}c_{2}},\frac{\left(1-c_{1}\right)M_{2}\left(t\right)}{1-c_{1}c_{2}}\right)$. The point $A_{1}$ indicates strong competitiveness for college education informatics innovation community $M_{1}$ diffusion of innovations in educational informatics than the college community $M_{2}$ of educational informatization innovation, after the diffusion and balance of the group education informatization innovation between the two universities, the field of education informatization innovation is occupied by the university group $M_{1}$, and the university group $M_{2}$ is squeezed out of the field of education informatization innovation; The situation is similar for $A_{2}$. The point $A_{3}$ indicates that the competitiveness of the diffusion of education informatization innovation between the two groups of universities is comparable, and the two sides fail to push each other out of the field of education informatization innovation, and the diffusion of education informatization innovation is finally in a state of coexistence.

## 3 Test analysis

Taking a number of colleges and universities in a certain region as the research object, the method of this paper is used to study the influence of various factors on the diffusion process of educational informatization innovation in colleges and universities. The region contains a total of two colleges and universities, and the relevant introductions of the two colleges and universities are shown in Table 2.

**Table 2:** Relevant introductions of universities.

University number	Investment in information infrastructure (ten thousand yuan)	Usage rate of online teaching platform (%)	Number of digital teaching resources (PCS)	Number of smart classrooms (rooms)	Number of informatization training (times/year)
1	500	95	1200	30	10
2	450	92	950	28	12

**Fig 2:**
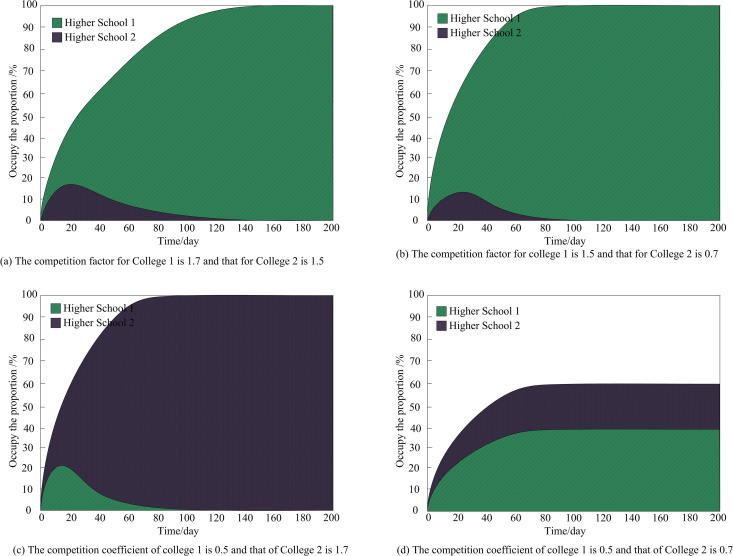
Diffusion process of university education informatization under the influence of different competition coefficients.

The competition coefficient reflects the diffusion ability of educational informatization innovation in universities. In order to analyze the influence of the competition coefficient on the diffusion process, we set the diffusion speed of educational informatization innovation of two colleges and universities as 0.5, and analyze the influence of different competition coefficients on the diffusion process of educational informatization innovation of the two colleges and universities in the region which are adopting or about to adopt informatization innovations. The results of the analysis are shown in Fig 2.

It can be seen from Fig 2(a) that when the competition coefficient of both universities is greater than 1, it indicates that the two universities have a greater inhibitory effect on each other’s education informatization innovation diffusion process. After fierce competition, university 1 with a higher competition coefficient has a competitive advantage. In about 140 days, the proportion of university 1 in the education informatization innovation field is 100%, and the education informatization innovation will be fully realized. It has a better effect of educational informatization innovation diffusion. However, the competition coefficient of university 2 is lower than that of university 1. In about 140 days, the proportion of university 2 in the field of educational informatization innovation is 0%, and it will completely withdraw from educational informatization innovation.

It can be seen from Fig 2(b) that when the sum of the two competition coefficients is greater than 1 and the competition coefficient of university 2 is less than 1, the final equilibrium state must be that university 1, which has a competitive advantage, fully realizes the innovation of educational informatization. In about 90 days, university 1 accounts for 100% of the total, that is, university 1 has a better diffusion effect of educational informatization innovation, and university 2 completely withdraws from educational informatization innovation.

It can be seen from Fig 2(c) that when the sum of the two competition coefficients is greater than 1 and the competition coefficient of university 1 is less than 1, the final equilibrium state must be that university 2, which occupies a competitive advantage and fully realizes the innovation of educational informatization. In about 90 days, university 2 accounts for 100% and has a better diffusion effect of educational informatization innovation, and university 1 completely exits educational informatization innovation.

It can be seen from Fig 2(d) that when the two competition coefficients are both less than 1, it means that both sides have less inhibitory effect on each other, and the final equilibrium state is that the two universities jointly realize educational informatization innovation, and the number of teaching disciplines that finally implement informatization innovation is affected by the competition coefficient; Universities with high competition coefficient 2 have strong competitiveness and good diffusion effect of educational informatization innovation. Their proportion in the field of educational informatization innovation is close to 60%. Comprehensive analysis shows that universities with higher competition coefficient have stronger competitiveness and better diffusion effect of educational informatization innovation.

**Fig 3:**
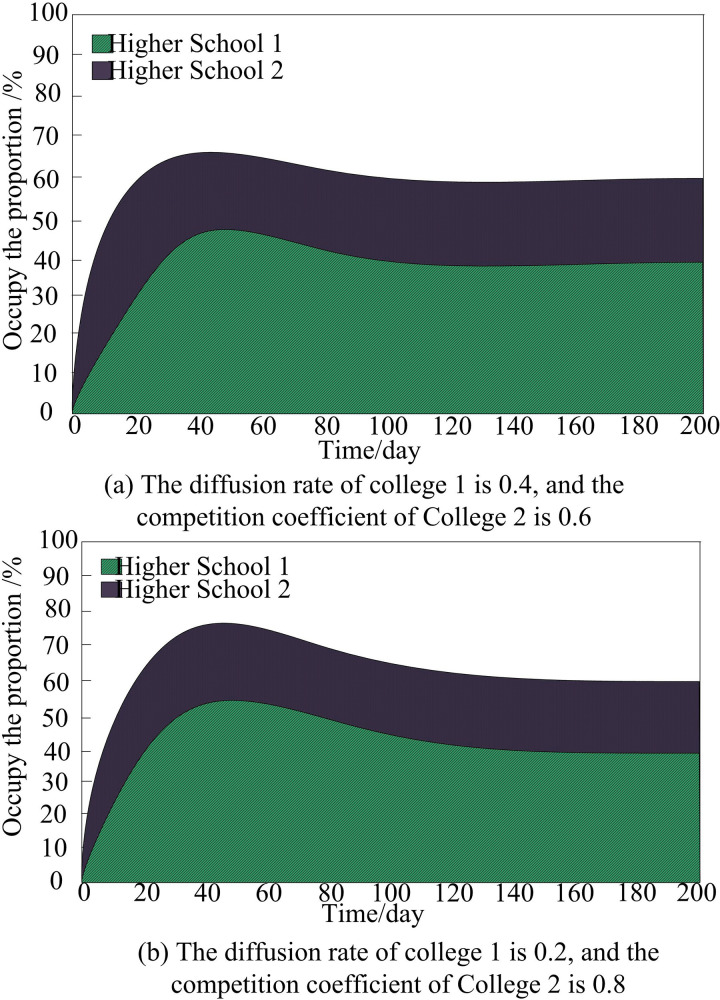
Diffusion process of university education informatization innovation under the influence of different diffusion speeds.

In order to analyze the influence of diffusion speed on the diffusion process of educational informatization innovation in colleges and universities, we choose the competition coefficient of college 1 as 0.5, and the competition coefficient of college 2 as 0.7, and analyze the diffusion process of educational informatization innovation in two colleges and universities under the influence of different diffusion speeds, and the results of the analysis are shown in Fig 3.

It can be seen from Fig 3(a) and 3(b) that in the process of innovation diffusion of university education informatization, the achievement of the final equilibrium state does not directly depend on the value of the diffusion speed, that is, the diffusion speed will not affect the final equilibrium of innovation diffusion of university education informatization. As a key variable, diffusion speed has a significant impact on the timing of the inflection point of educational informatization innovation diffusion.

When the difference in the diffusion speed of education informatization innovation between two universities is small, it will directly affect the inflection point of diffusion. Advantageous colleges and universities, i.e., those that can adopt and implement education informatization innovations faster and more effectively, tend to obtain higher inflection point values. The result means that they can realize the improvement of education informatization level earlier, enjoy the improvement of teaching and management efficiency brought by the innovation, and thus occupy a favorable position in the competition. 2 colleges and universities diffusion speed of the gap is larger, the existence of the advantage of colleges and universities 2 of the diffusion of the inflection point of the value of the larger, that is, colleges and universities 2 of the education informatization innovation can be adopted and implemented more quickly and effectively, which means that colleges and universities 2 of education informatization innovation to be able to realize the improvement of the level of informationization. It means that university 2 can achieve the improvement of education informatization level earlier and enjoy the improvement of teaching and management efficiency brought by the innovation, so as to occupy a favorable position in the competition. At the same time, the time needed for the two universities to reach the equilibrium state of diffusion of education informatization innovation is also prolonged accordingly, which is because the universities with slower diffusion speed need longer time to catch up and the whole equilibrium state needs to be waited for until all the participating universities have reached a certain level of education informatization. The whole equilibrium state needs to wait until all participating universities have reached a certain level of education informatization. Therefore, it is of great significance to reduce the speed gap of diffusion of education informatization innovation among universities to accelerate the informatization process of the whole education system.

In summary, in order to promote the balanced development of education informatization, it is necessary to narrow the gap in the speed of diffusion of education informatization innovations among colleges and universities, and to improve the effect of diffusion of education informatization innovations in colleges and universities and their education informatization capabilities.

When analyzing the impact of innovation diffusion of university education informatization, Cronbach’s α coefficient and Composite Reliability (CR) are used as important statistical indicators to evaluate the reliability of measurement indicators, so as to ensure the internal consistency and reliability of the collected data. The Cronbach’s α value is usually used to measure the internal consistency of the scale. The closer the value is to 1, the higher is the internal consistency. The Cronbach’s α value range is [0.789, 0.855] (Cronbach’s α standard is > 0.7). The standardization factor load reflects the correlation strength between items and their variables, which is generally required to be greater than 0.7 to ensure the effective contribution of items to variables. Table 3 shows the impact results of innovation diffusion of university education informatization.

**Table 3:** Influence results of innovation diffusion of informatization in higher education.

Variables that affect the result type	Item	Standardized factor load	Cronbach’s α
Teaching content	1. Course content update	0.82	0.9
	2. Resource integration	0.79	
	3. Share resources	0.85	
Teaching form	1. Multimedia teaching	0.77	0.88
	2. Distance learning	0.81	
	3. Personalized teaching	0.76	
Teacher role	1. Role transformation	0.8	0.85
	2. Information literacy improvement	0.75	
	3. Lifelong learning	0.83	
Student role	1. Increased initiative	0.84	0.92
	2. Improve independent learning ability	0.81	
	3. Diversify	0.78	
Educational efficiency	1. Time efficiency is improved	0.76	0.84
	2. Improved resource utilization efficiency	0.8	
Educational equity	1. Geographical restrictions are broken	0.79	0.87
	2. Concern of vulnerable groups	0.82	

It can be seen from Table 3 that the Cronbach’s α value of the teaching content variable is higher than 0.7, and the standardized load factor of its entry is also higher than 0.7, so it can be considered that the diffusion of university education informatization has achieved stable and significant impact results in the renewal of teaching content, resource integration and resource sharing. It shows that the informatization of college education not only promotes the digitization and multimedia of traditional teaching content, but also enables teaching content to keep pace with the times and reflect the cutting-edge trends of disciplines in a timely manner. The rapid updating ability provides students with more abundant and cutting-edge learning resources, which helps to cultivate their innovative thinking and practical ability. By means of informatization, colleges and universities can more effectively integrate high-quality educational resources inside and outside the school, break information silos, and realize resource sharing. This can not only improve the efficiency of resource utilization, but also provide students with more diversified learning ways, and promote the integration and innovation of interdisciplinary knowledge.

The Cronbach’s α value of the teaching form variable is higher than 0.7, and the standardized load factor of its entry is also higher than 0.7, so it can be considered that the diffusion of college education informatization has achieved stable and significant impact results in multimedia teaching, distance teaching and personalized teaching. It shows that multimedia teaching can greatly enhance students’ interest and enthusiasm in learning with its intuitive, vivid, large amount of information and other characteristics. The introduction of distance learning and personalized teaching breaks the time and space constraints and provides more flexible and personalized learning programs for students with different learning styles and ability levels. The informatization of education has also given birth to new teaching models such as flipped classroom and mixed teaching. These models emphasize the dominant position of students, encourage independent inquiry and cooperative learning, and help cultivate students’ critical thinking and problem-solving ability.

The Cronbach’s α value of teacher role variable is higher than 0.7, and the standardized load factor of its item is also higher than 0.7, so it can be considered that university education informatization diffusion has achieved stable and significant impact results in role transformation, information literacy improvement and lifelong learning. It shows that under the background of educational informatization innovation, teachers need not only to have solid professional knowledge, but also to master the application ability of information technology, so as to better use informatization means to carry out teaching. The innovation of educational informatization requires teachers to constantly update their knowledge structure and teaching concepts to adapt to the rapidly changing educational environment. Therefore, lifelong learning has become an important trend of teachers’ professional development. Through continuous learning and practice, teachers can constantly improve their teaching ability and provide students with higher quality education services.

The Cronbach’s α value of the student role variable is higher than 0.7, and the standardized load factor of its entry is also higher than 0.7, so it can be considered that the diffusion of educational informatization in colleges and universities has achieved stable and significant results in enhancing initiative, as well as improving autonomous learning ability and diversified development. The education informatization innovation provides more opportunities and platforms for students to learn independently, and stimulates their learning interest and initiative. Students can choose learning content and learning methods according to their own interests and needs to achieve personalized learning. In the information innovation environment, students need to learn how to obtain information, process information and use information to solve problems. The cultivation of this ability not only helps students improve their academic performance during school, but also lays a solid foundation for their future lifelong learning and career development. The innovation of educational informatization also provides students with more diversified learning resources and ways, which helps them develop their own interests and specialties. This diversified development not only enriches students’ learning experience, but also promotes their comprehensive quality.

The Cronbach’s α value of the education efficiency variable is higher than 0.7, and the standardized load factor of its entry is also higher than 0.7, so it can be considered that the diffusion of university education informatization has achieved stable and significant impact results in improving time efficiency and resource utilization efficiency. Education informatization innovation significantly improves education efficiency by optimizing teaching process and improving resource utilization efficiency. This is not only helpful to alleviate the shortage of educational resources, but also provides students with a more efficient and convenient learning experience.

The Cronbach’s α value of the education equity variable is higher than 0.7, and the standardized load factor of its entry is also higher than 0.7. Therefore, it can be concluded that the diffusion of educational informatization in colleges and universities has achieved a stable and significant effect in breaking through regional restrictions and focusing on vulnerable groups. The innovation of educational informatization has broken the regional restrictions and class barriers, making more students have the opportunity to accept high-quality educational resources. At the same time, through measures such as paying attention to vulnerable groups and providing differentiated support, educational informatization innovation can also help to narrow the educational gap and promote the realization of educational equity.

The comprehensive analysis shows that after the expansion of the innovation of college education informatization, stable and reliable impact results have been achieved in the aspects of teaching content, teaching form, teacher’s role, student’s role, education efficiency, and education fairness.

## 4 Conclusion

The diffusion process of educational informatization innovation in colleges and universities is affected by many factors, and different colleges and universities will also be affected by other colleges and universities in the process of educational informatization innovation. In order to reveal the diffusion process of educational informatization technology in the university environment, this paper studies the diffusion process of educational informatization innovation in colleges and universities based on Lotka-Volterra model. Research finds:

(1) The ideal equilibrium state of competitive diffusion is shown as follows: the competition coefficient affects the process of diffusion of educational informatization innovations in colleges and universities, and colleges and universities with higher competition coefficients have better results in the diffusion of educational informatization innovations.
(2) The impact types of the diffusion of educational informatization innovation in colleges and universities are various, mainly including educational efficiency and educational equity. The Cronbach’s α value of each impact type variable is higher than 0.7, indicating that the impact of the diffusion of educational informatization in colleges and universities in these aspects is stable and reliable.

The research content of this paper helps colleges and universities to understand the characteristics and laws of the diffusion process of educational informatization innovation, and provides a quantitative decision-making basis for the educational system of colleges and universities, the government and other departments to promote the construction of educational informatization. According to the conclusion of the study, corresponding countermeasures and suggestions are put forward: Firstly, in the process of educational informationization construction, the cooperation and promotion relationship between universities should be given full play, the inhibition of competition should be weakened, and the information collaborative innovation cultivation, policy coordination and coordination between similar universities should be actively promoted.; Secondly, the role of higher education system and government departments in promoting the construction of higher education informatization should be given full play. According to the different stages of university informationization innovation, the slow development of university education informationization should be actively supported in terms of financial support, policy support, team support, and infrastructure construction; It can provide long-term development guarantee measures in terms of enhancing comprehensive strength, strengthening industry, university and research institutes, establishing information data governance, improving the specific application system of information innovation system, and information system and mechanism.

Although this study conducted an in-depth analysis of the diffusion process of information technology innovation in higher education based on the Lotka Volterra model, there are still certain limitations. Firstly, in terms of data collection, due to the involvement of multiple complex factors in the diffusion process of information technology innovation in higher education, data acquisition is difficult, which may result in incomplete and inaccurate data, thereby affecting the accuracy of research results. Secondly, during the model construction process, although the Lotka Volterra model provides an effective theoretical framework for analyzing innovation diffusion, it cannot fully cover all the complex mechanisms and dynamic changes in the process of innovation diffusion in higher education informationization, including cultural differences, regional differences, and other factors that are not fully reflected in the model. In addition, this study mainly focuses on the impact of competition and cooperation among universities on the diffusion of educational informatization innovation, while other potential influencing factors such as internal management mechanisms and individual teachers’ innovation willingness have not been explored in depth. Therefore, based on the limitations of this study, future research can further improve data collection methods, broaden data source channels, enhance the comprehensiveness and accuracy of data, in order to more accurately reveal the inherent laws of the diffusion process of information technology innovation in higher education. Secondly, the Lotka Volterra model is improved and expanded by introducing more factors that can reflect the actual diffusion of information technology innovation in higher education, including cultural and regional factors, to make the model more in line with real-life situations. At the same time, in-depth research should be conducted on the impact of internal management mechanisms and individual innovation willingness of teachers in universities on the diffusion of educational informatization innovation, as well as the interaction mechanism between these factors and other factors, in order to provide more comprehensive and in-depth decision-making basis for the construction of educational informatization in universities. In addition, comparative research can be conducted across regions and types of universities to analyze the differences and similarities in the diffusion of educational informatization innovation among different regions and types of universities, providing reference for formulating more targeted policies for educational informatization construction.
